# Calibration, Sensitivity and Uncertainty Analysis of Complex Ecological Models—A Review

**DOI:** 10.1111/ele.70375

**Published:** 2026-04-26

**Authors:** Anne‐Kathleen Malchow, Florian Hartig

**Affiliations:** ^1^ Theoretical Ecology University of Regensburg Regensburg Germany

**Keywords:** Bayesian inference, informal likelihood, inverse modelling, parameter estimation, predictive uncertainty, sensitivity analysis, simulation models, uncertainty propagation

## Abstract

Ecologists increasingly use complex models to predict and understand ecological systems and their responses to external drivers or anthropogenic pressures. An ongoing challenge in this context is quantifying and reducing uncertainty in model inputs, parameters and structure and understanding their implications for model predictions. Three major methodological fields have emerged in this context: sensitivity analysis, uncertainty analysis and model inversion or calibration. While these three methods are an integral part of any modelling or forecasting process, the corresponding literature is often scattered, and distinct terminology and definitions are used in different methodological and scientific contexts. Here, we review and connect these three fields and discuss best practices for their practical implementation with a focus on complex ecological models. We classify relevant types of uncertainty, discuss the complementary roles of sensitivity and uncertainty analyses, give an overview of available calibration methods and emphasize the importance of effective communication of uncertainty. We conclude that using state‐of‐the‐art methods for understanding model behaviour as well as consistently accounting for all uncertainties is essential for correctly understanding model predictions and thus forms the basis for a responsible use of models in ecological decision making.

## Graphical abstract

We provide a unified framework connecting sensitivity analysis, uncertainty analysis and model inversion or calibration, discuss their complementary roles in understanding and reducing uncertainty in complex ecological models, and offer practical recommendations for their implementation.

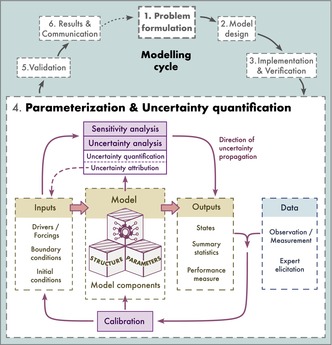

## 1 Introduction

In the ecological sciences, complex models are increasingly used to predict ecosystem dynamics and their responses to external drivers (e.g., Bonan and Doney 2018; Geary et al. 2020; Urban et al. 2016). It has been argued that complex mechanistic models will not only promote our understanding of key ecosystem mechanisms and pressures, such as habitat degradation and climate change, but are also essential for guiding ecological management (e.g., Evans et al. 2013; Newbold 2018; Zurell et al. 2022). However, as stressed by many recent articles on the emerging paradigm of ‘predictive ecology’ (e.g., Mouquet et al. 2015; Petchey et al. 2015; Schuwirth et al. 2019; Dietze et al. 2018), quantifying and reducing uncertainties of complex ecological models remains a key challenge.

Model complexity, as the term is used in this review, implies that a model depends on a multitude of components (e.g., structure, states or parameters) and inputs (e.g., drivers, initial and boundary conditions), and that modifying its components and inputs can have non‐trivial and difficult‐to‐anticipate effects on the model's behaviour. In this situation, advanced methods are needed to understand how uncertainties in model components or inputs affect model outputs (‘*forward problem*’); or, conversely, how information on model outputs can be propagated back to reduce uncertainty in model components and inputs (‘*inverse problem*’). For later reference of these and other technical terms, see our glossary (Box 1).

BOX 1 **Glossary.**

- *Calibration*: The process of choosing parameter values such that model predictions match observations. A special case of inverse modelling.
- *Curse of dimensionality*: The phenomenon that the number of computations needed to adequately cover the input space of a model increases exponentially with the number of input factors that are varied (see Box S1).
- *Equifinality*: The situation in which different parameterizations or candidate models fit equally well to the data.
- *Forward modelling or forward problem*: The process of deriving model outputs from model assumptions and inputs. Includes running scenarios, but also sensitivity analysis and uncertainty propagation.
- *Inverse modelling or inverse problem*: The process of inferring a model's components (parameters, structure) or inputs from its outputs. Includes calibration, model comparison or selection, and data assimilation.
- *Model*: A function M that assigns inputs *x* to outputs *y* = *M*(*x*; *p*, φ) with the purpose of representing the behaviour of a real system. Within a model, we distinguish the model structure *p* and model parameters φ. If a fixed model structure is to be parameterized, it may also be referred to as *M*(*x*; φ) = *f*_*p*_(*x*; φ). A model can be represented by mathematical formulas (e.g., partial differential equations) but also by (stochastic) computer algorithms (e.g., individual‐based models).
- *Model comparison*: Assessment of the relative support for multiple candidate models, usually through quantification of the goodness‐of‐fit.
- *Model components*: Internal variables such as parameters or structure that determine model behaviour.
- *Model inputs*: External variables such as drivers/forcings, initial conditions, or boundary conditions that affect the model's predictions.
- *Model outputs*: All model quantities whose values are determined by running the model, for example, abundance patterns, population structure, community composition.
- *Parameterization*: (1) a set of values to assign to model parameters, or (2) the process of determining these values (can involve direct and/or inverse parameterization).
- *Projection*: A ‘what‐if’ model prediction conditional on some inputs that are not assigned an explicit probability.
- *Prediction*: The output of a parameterized model (potentially including uncertainties) under probabilistic (future) conditions.
- *Sensitivity analysis*: Evaluates how strongly a model output responds to changes in an input factor, for example, a driver/scenario, parameter value, or model structure.
- *Uncertainty*: The lack of sufficient information to describe a model component or input with a single value.
- *Uncertainty analysis*: Identification and representation of (ideally all) uncertainties present in a model, following three steps:
  ○ *Uncertainty quantification*: Characterization and quantification of uncertainty in model components and inputs.
  ○ *Uncertainty propagation*: Quantification of outcome uncertainty by forwarding the identified uncertainties to model projections.
  ○ *Uncertainty attribution*: Quantification of the individual contributions of each input uncertainty to the outcome uncertainty.

Among these approaches, three broad classes of methods can be distinguished: sensitivity analysis, uncertainty analysis and model inversion. *Sensitivity analysis* quantifies the response of model outputs to changes in model components or inputs, either locally or globally (e.g., Saltelli et al. 2006). Closely related, *uncertainty analysis* quantifies the total uncertainty in a model output and attributes it to the various input uncertainties (Helton and Davis 2003). Lastly, *model inversion* encompasses all methods that infer the structure, parameters or states of a model by comparing its predictions to observed data. Inversion methods, such as parameter *calibration* or data assimilation, can be broadly divided into two groups: (i) informal calibration approaches, such as pattern‐oriented modelling (POM, see Grimm et al. 2005; Wiegand et al. 2003; see Semeniuk et al. 2012 for an ecological example) or generalized‐likelihood uncertainty estimation (GLUE, see Beven and Binley 1992); and (ii) statistical approaches that are typically based on likelihood or Bayesian inference (e.g., Ellison 2004; Kattwinkel and Reichert 2017). Common to both groups is the use of various search algorithms for finding the model version that provides the ‘best fit’ to the data, most notably optimization and MCMC algorithms.

Together, sensitivity analysis, uncertainty analysis and inversion or calibration methods constitute a framework for the identification, quantification, and reduction of different types of uncertainty, as well as the analysis of their sources and interactions. From a forward‐looking perspective, a model's final prediction uncertainty is determined by two main factors: (1) the amount of uncertainty contributed by the different model components and inputs (exposure) and (2) the respective sensitivities of the model to these factors (susceptibility). Conversely, from an inverse perspective, observational data on a model prediction can most easily reduce uncertainty in model components or inputs that are sensitive to that prediction. Combining forward and inverse uncertainty propagation can thus provide important insights into a model's behaviour and help to understand, propagate and reduce uncertainties arising from the various model components.

Unfortunately, such a unified view in which sensitivity analysis, uncertainty analysis and model inversion are used synergistically is often clouded by the diversity of methodological options that exist for each step, as well as by the divergent terminology that is used in different scientific fields. Most reviews on these topics focus on a single methodological approach and/or on specific model types. For instance, comprehensive overviews of sensitivity analysis practices are given in Pianosi et al. (2016), Saltelli et al. (2006), and Razavi et al. (2021). Uncertainty analysis methods differ for parametric uncertainty (e.g., Reimer et al. 2022), structural uncertainty (Refsgaard et al. 2006) or deep learning models (Abdar et al. 2021). Looking only at parametric uncertainty, the calibration of stochastic models is reviewed, for example, in Hartig et al. (2011); and the calibration of state‐space models in Auger‐Méthé et al. (2021) or, for highly nonlinear models, in Fasiolo et al. (2016). A broad overview covering all three tasks is given in Zhang et al. (2020), but with a focus on models in the field of engineering that can be expressed as systems of (partial) differential equations. Moreover, the appropriate communication of uncertainties (Spiegelhalter 2017) is often not part of these technical reviews.

**FIGURE 1:**
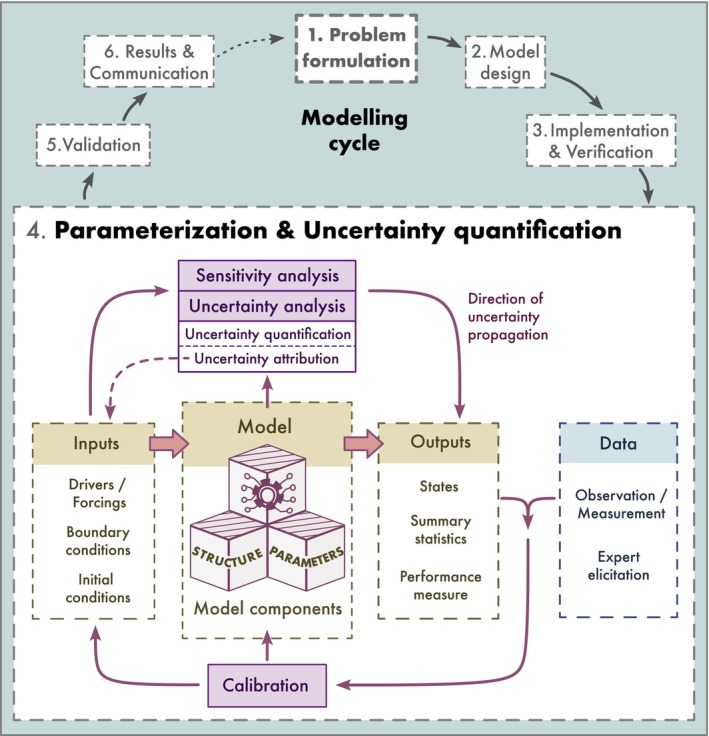
Location and importance of sensitivity analysis, uncertainty analysis and model inversion or calibration (violet boxes) in the context of the full cycle of ecological model development.

The goal of this paper is thus to provide a synergistic summary of sensitivity analysis, uncertainty analysis and model inversion or calibration and place them into a common framework for dealing with uncertainty in complex ecological models (Figure 1). We start by discussing what we mean by a model and provide a classification of uncertainties. We then provide an overview of forward and inverse methods for the quantification, reduction, and attribution of these uncertainties. The application of each technique is discussed in the context of complex ecological models, for which we give practical recommendations and guidance. We conclude by discussing the effective communication of uncertainty.

## 2 Models and their Uncertainties

Ecologists use a variety of models to understand and predict eco‐evolutionary patterns at specific organizational, spatial and temporal scales (Cabral et al. 2017; Getz et al. 2018). These models may be formulated as mathematical equations or computer code; they may act on continuous or discrete scales; and they may be deterministic or stochastic. Examples include general ecosystem models (Harfoot et al. 2014; Töpper et al. 2025), dynamic (global) vegetation models (Sato et al. 2007; Smith et al. 2014), macroevolutionary biodiversity models (Hagen et al. 2021), spatial population models (Bocedi et al. 2021), individual‐based models (Railsback and Grimm 2019) or resource‐consumer models (Rosenbaum and Rall 2018). Ecological models can further be divided into static and dynamic models: static models such as correlative species distribution models (SDMs) may implicitly represent causal mechanisms, but they do not explicitly describe the temporal sequence that led to an observed pattern (cf. Dormann et al. 2012). Dynamic models, on the other hand, such as movement models or ecosystem models, have time‐dependent internal states that are (iteratively) advanced from given initial conditions and based on given boundary conditions (drivers).

Complex models and their predictions consist of and are influenced by a number of quantities. In this review, we distinguish two main groups of such quantities: model components and model inputs. *Model components* (see also Glossary), such as structure, states and parameters, are the internal parts of a model that represent the implemented ecological processes. *Model inputs*, in contrast, are external factors. In ecology, they typically consist of dynamic drivers or forcings (e.g., climate); boundary conditions (e.g., soil, topography) and, for dynamic models, initial conditions (e.g., initial population or size structure).

The process of ecological model development is often characterized as a cycle (Figure 1; see also Schmolke et al. 2010). Its first two steps are (1) the problem formulation and (2) the model design, where the model components are determined (Bodner et al. 2020; Jakeman et al. 2006). Once an adequate design is determined, the model is implemented and verified in step (3) to check whether it behaves as conceptualized. Here, two types of errors can occur: technical errors (e.g., in the programming) and logical errors in the algorithmic implementation (Rykiel 1996). To verify that the model performs as expected, a sensitivity analysis can be helpful, but it can also be postponed to the next step. In step (4), the model is parameterized and the associated uncertainties are quantified. Parameters can be determined either directly from available information or by calibration to observed data. How uncertainties affect model predictions can be assessed in an uncertainty analysis. In the validation step (5), the predictions of the parameterized model are tested against independent reference data to assess the predictive performance of the calibrated model. In step (6), the final model is used to make predictions and communicate them.

### 2.1 Classifying Uncertainties in Model Development

Every model has systematic errors and uncertainties. This is particularly true in ecology, where complex emergent phenomena are modelled that require simplifying assumptions. Understanding the nature of the involved uncertainties is critical for many reasons, including decision‐making. As Dietze et al. (2018) put it: ‘Uncertainty is at the core of how people evaluate risk and make decisions’.

A first step in an uncertainty assessment is to analyse where uncertainties exist in a model (see Bevan (2022) for a comprehensive review). Here, we follow Walker et al. (2003), who classify uncertainties based on three properties: location, level, and nature of uncertainty.

The part of the model where the uncertainty is manifest is known as its *location*: *Structural or model uncertainty* encompasses uncertainties arising from model design and implementation. This includes choices relating to conceptualization and process assumptions, as well as possible coding or hardware errors. *Parameter uncertainty* refers to uncertainty in the adjustable parameters of a model. *Input uncertainty* describes uncertainty in the conditions for which a prediction is to be made, such as drivers/forcings, initial conditions and boundary conditions. *Model outcome uncertainty* is the uncertainty around a model output of interest, which arises as a result of all other uncertainties.

How well an uncertain quantity can be described under incomplete knowledge is called the *level* of uncertainty (Walker et al. 2003). It ranges from recognized ignorance (known unknowns) to determinism (i.e., precise knowledge) and determines which methods for uncertainty analysis and calibration are available. In practice, modellers often have a certain level of information on parameter or structural uncertainty, such as a list of plausible values, ranges or full probability distributions for parameters. However, some processes may not even be considered by the modeller (‘unknown unknowns’, e.g., Walker et al. 2003). Such unrecognized uncertainty is often referred to as total ignorance, deep uncertainty or surprises (Parker and Risbey 2015; Spiegelhalter and Riesch 2011; Walker et al. 2003).

Lastly, uncertainty is often classified as aleatoric or epistemic, describing the *nature* of uncertainty (Walker et al. 2003). Aleatoric uncertainty stems from the inherent stochasticity of a system that cannot be further reduced at the resolution of the modelled processes. In ecological models, such uncertainty could be expressed, for example, by a stochastic movement or mortality process. Epistemic uncertainty, in contrast, is due to insufficient or inadequate knowledge about the model components or inputs and is therefore, in principle, reducible by appropriate methods and data.

## 3 Sensitivity Analysis

A central tool to explore the effects of model uncertainties is *sensitivity analysis* (*SA*). Model *sensitivity* describes how strongly model outputs change in response to changes in model components or inputs (both are referred to as *input factors* in the context of sensitivity analysis). The process of assessing model sensitivities is called sensitivity analysis.

**FIGURE 2:**
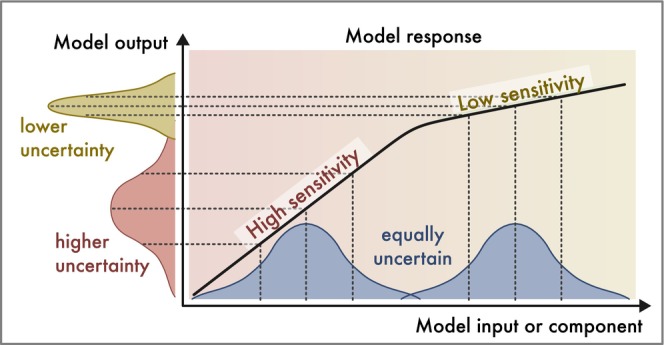
The sensitivity of a model can be defined as the slope of the relationship between input factors and model outputs, or, more technically: The derivative of a model output with respect to an input factor. Input factors may include parameters, structure, initial conditions or drivers of the model. Note that sensitivity is a property of the model and can thus be assessed without knowledge of input uncertainties. However, sensitivity is connected to output uncertainty because it controls how given input uncertainties propagate to output uncertainties: In regions of higher (lower) sensitivity, the same amount of input uncertainty results in higher (lower) output uncertainty (visualization follows Loucks and van Beek 2017).

In principle, sensitivity is a property of model structure and can be studied independently from uncertainties. In practice, however, many SA methods calculate sensitivities over the uncertainty range of input factors, thus benefiting from a preceding *uncertainty analysis* (*UA*). Broadly speaking, output uncertainty then emerges from a model's sensitivity to an input factor times this input factor's uncertainty (Figure 2). Therefore, the line between UA and SA is not always clear (e.g., Saltelli et al. (2006) view the attribution of output uncertainty to different input uncertainties as part of an SA, thereby combining UA and SA). We nevertheless recommend keeping a clear conceptual distinction between the quantification of sensitivity as a model property (SA) and the study of the effect of input uncertainties on output uncertainties (UA).

The choice of ‘target’ for an SA, that is, the model output for which sensitivities are to be calculated, depends on its purpose. In ecology, the typical target is an output of primary interest to the study, such as biomass or species diversity. This sensitivity target will often also be subject to a downstream uncertainty analysis (see Section 4). When models are built for policy or management, a SA may also target management recommendations that are calculated from the model. In addition to gaining insight into model behaviour, SA is often recommended to verify that a model conforms to biological expectations or to identify unimportant input factors that hold potential for model simplification. For this purpose, targets can be chosen for which ecological plausibility can be evaluated. Finally, in the context of model uncertainties, SA can help prioritize uncertainty reduction or assess which parameters are likely to be identifiable by inverse calibration, since more sensitive parameters tend to be more readily constrained by calibration data (Monsalve‐Bravo et al. 2022). In this case, the SA target must be the calibration target that measures the model's fit to the data (see Section 5).

### 3.1 Algorithms for Sensitivity Analyses

The full information about a model's sensitivity is contained in the response surface that maps the space of input factors onto model outputs. With a single input factor, such a response is easy to calculate and visualize (e.g., Figure 2), but with more input factors, the number of computations necessary to fully cover the response surface typically increases exponentially (known as the *curse of dimensionality*, see Box S1), which makes a full coverage and visualization of the response surface challenging.

SA methods circumvent this problem either by calculating and summarizing the model response at a specific point (local SA) or by calculating average responses across a specific region of the input space (global SA). Conceptually, these methods have strong parallels with the currently emerging field of explainable AI (xAI), which also aims at finding simplified approximations of complex machine learning or deep learning models (Ryo et al. 2021). The available SA methods are often broadly classified according to two properties (Table S1): (1) The dimensionality of the sampled (sub‐)space of input factors, which orders methods from local to global. (2) The sensitivity measure used (e.g., derivatives, finite differences or variance partitioning). We briefly introduce both concepts and their corresponding methods. More comprehensive overviews of SA methods are given in Borgonovo and Plischke (2016), Norton (2015), Pianosi et al. (2016) and Frey and Patil (2002), and comparisons of different global SA methods were conducted by Confalonieri et al. (2010) and Cosenza et al. (2013). Many of these methods are implemented in the R package ‘sensitivity’ (Pujol and Iooss 2015).

**FIGURE 3:**
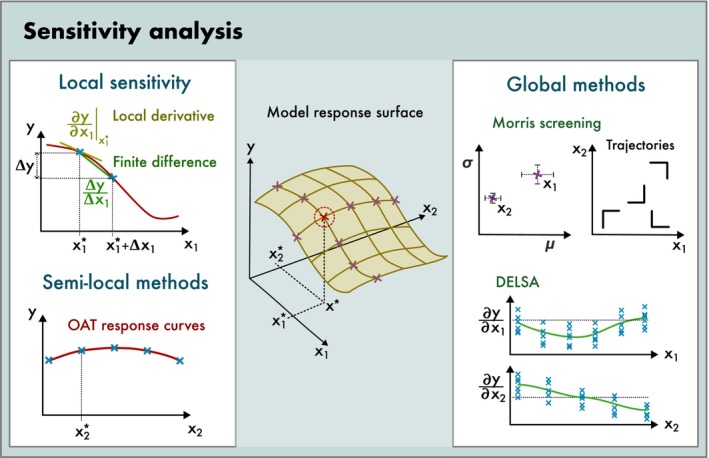
Sensitivity analysis methods summarize the shape of the full model response surface (middle). A local sensitivity analysis (top left) quantifies the derivative (multivariate, potentially approximated by finite differences) at a reference point. Semi‐local methods (bottom left) calculate local sensitivities across several points in the input space, for example by calculating one‐factor‐at‐a‐time (OAT) response curves. Global methods (right box) vary multiple or all inputs together within given boundaries and thus also capture the effects of input interactions. Illustrated are two examples of slope‐based global methods: Morris screening (top right) and DELSA (bottom right).

The dimensionality of the sampled space determines the ability of the SA to resolve nonlinearities and interactions (Figure 3). *Local SA* methods calculate sensitivity at one reference point x*, typically the default parameterization of the model. While this approach is easy to implement, it cannot detect nonlinearities or interactions in the larger input space around x*. *Semi‐local SA* methods such as one‐(factor‐)at‐a‐time (OAT; also known as mono‐factorial) vary one input factor across a wider range while all other input factors remain fixed at the reference x*. Therefore, semi‐local SA can show the effect of nonlinearities (as in Figures 2 and 3) but cannot resolve interactions between multiple factors. *Global SA* methods vary all input factors simultaneously within specified ranges and can thus account for both nonlinearities and interactions among input factors.

The lower and upper bounds for a global SA can be chosen ad hoc but can also be determined from an uncertainty quantification. In the latter case, global sensitivity indices can be interpreted as an estimate of how different factors contribute to output uncertainty (see next Section 4).

SA methods can be further classified by how sensitivity is measured. We primarily distinguish between metrics that target the slope of the response surface (akin to our intuitive definition of sensitivity in Figure 2) and metrics that evaluate the variance explained by an input factor.

SA metrics that evaluate the slope include *derivative‐based SA* and *finite‐differences‐based SA*. The former quantify the slope at a local point, either analytically or via numeric approximation, whereas the latter quantify the slope over a larger interval (e.g., ±10% of the reference value). Both methods can easily be expanded to interactions by calculating higher‐order derivatives. Because derivative‐based SA is the only fully local method, it is often called ‘local’ SA. However, both derivative‐based and finite‐differences‐based sensitivities can also be used in a global SA by averaging them over a larger space. For example, Morris screening calculates finite differences along random trajectories in input space (Figure 3; Morris 1991). Other examples are global–local SA methods that use a global sampling and a local sensitivity measure, such as DGSM (derivative‐based global sensitivity measures; Kucherenko et al. 2009) or DELSA (distributed evaluation of local sensitivity analysis, Figure 3; Rakovec et al. 2014).

*Variance‐based SA methods* attribute the output variance to the variances of different input factors, similar to an ANOVA. This approach makes most sense for a global SA, where many input factors are varied simultaneously. One of the most widely used methods of this class is the Sobol' method (Sobol’ 1990; Sobol' 2001), which decomposes the total output variance into first‐order (main) effects and higher‐order (interaction) effects.

A separate strategy to calculating sensitivities is approximating the response of a complicated model by a simpler model (also called surrogate model, meta‐model or emulator) for which sensitivities can be easily calculated. For example, if a regression model is used as a surrogate, its effect sizes and their confidence intervals can be directly interpreted as sensitivities. If a machine learning (ML) model, such as a random forest or an artificial neural network, is the surrogate, their respective xAI can be used to calculate sensitivities (e.g., Harper et al. 2011; Oberpriller et al. 2022).

A limitation of many methods for SA is that they require numeric input factors, which limits their use for exploring alternative model assumptions. However, some specialized methods exist to perform structural sensitivity analysis (e.g., Adamson and Morozov 2014).

### 3.2 Recommendations for Ecological Modellers

Many guidelines for SA advise that local SA is unreliable and thus only global SA should be used (e.g., Saltelli et al. 2006). We disagree with this viewpoint. We recommend that at an early stage in a modelling project, an OAT SA should be conducted to generate univariate response curves for each input factor (Figure 3). Despite its shortcomings, our experience is that this method provides valuable visual feedback about model behaviour and can act as an additional step of model verification (for an ecological example, see e.g., Appendix of Malchow et al. 2024).

While it is not yet standard practice in ecological modelling, we recommend conducting a global SA at an advanced stage of every modelling project. For fully‐fledged examples in ecological modelling, see Harper et al. (2011), Huber et al. (2018) and Oberpriller et al. (2022). Ideally, this is done after uncertainties have been quantified, so that these can be considered in the global SA. Which SA method to choose depends on computational resources; whether direct (first order, second order, …), total (first and higher order combined) effects, or both, are needed; and on whether modellers want to examine how sensitivity changes across the input space. The correlations between input factors can influence the choice of method as well—some global methods require uncorrelated inputs (e.g., Sobol’ and FAST), whereas others can also handle correlated inputs (e.g., ALE plots). An overview of these and other SA methods and their properties is given in Table S1.

Although scalability to high‐dimensional parameter spaces is an important design goal for advanced SA methods, the number of model evaluations needed for stabilizing the outputs of a global SA may still scale unfavourably with the number of model dimensions. The exact scaling relationship usually depends on the method and the model under consideration (cf. Box S1), but as a rule of thumb, SA for models with more than 20 input factors require more careful methodological consideration. Computational effort can sometimes be reduced by screening for the most sensitive factors prior to performing a global SA (e.g., shown in Janse et al. 2010). In any case, the convergence of the applied SA method should be carefully checked (Sarrazin et al. 2016).

An additional point to consider, especially for runtime‐intensive ecological models, is the reusability of the computed samples. Here, global methods that rely on stratified random sampling in the input space (such as linear regression, ANOVA or ML approaches, see e.g., Oberpriller et al. 2022) can be preferable to methods with specialized sampling strategies (such as the Sobol' method or Morris screening) because the generated computations have a greater potential to be reusable for other analyses, such as inference or the calculation of surrogate models.

A final recommendation relates to the calculation of global sensitivity indices: many functions and packages allow scaling those indices to the ranges of the input space. If input ranges are based on an *uncertainty quantification*, scaled sensitivities should be interpreted as indices of *uncertainty attribution* (see Section 4) rather than sensitivities. Whether scaled or unscaled indices are more appropriate depends on the goal of the analysis, but the distinction should be clearly communicated to avoid confusion.

## 4 Uncertainty Analysis

### 4.1 Goal and Scope of Uncertainty Analysis

**FIGURE 4:**
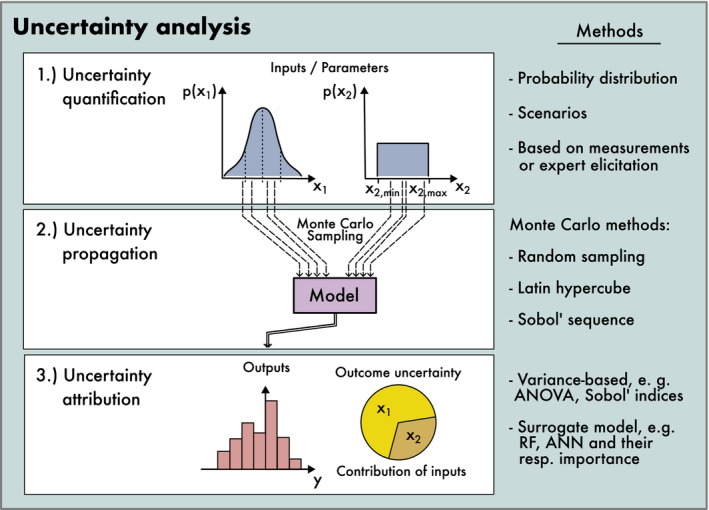
A complete uncertainty analysis consists of three steps, each of which has different methods available. (1) First, all uncertainties in model inputs and components are identified and quantified, ideally as probability distributions. (2) Then, values from the space of possible options are sampled and propagated through the model, creating a probabilistic or possibilistic distribution of model outcomes, depending on the level of input uncertainties. (3) Finally, the resulting outputs can be analysed to attribute the contributions of different input uncertainties to the output uncertainty.

Uncertainty analysis (UA) and sensitivity analysis (SA) are closely related, but not identical. The crucial distinction is that sensitivity is a property of the model that exists independent of the input uncertainty, whereas an UA measures the consequences of input uncertainty and sensitivity on model outputs. The process of an UA can be broken down into three steps (Figure 4): (1) uncertainty quantification, (2) uncertainty propagation and (3) uncertainty attribution. This pipeline forms the basis for robust management in ecology, as it allows assessing the range of plausible outcomes and tail risks (worst‐case outcomes).

### 4.2 Uncertainty Quantification

*Uncertainty quantification* refers to the identification and characterization of uncertainties in model inputs or components. This can be done by direct measurement and observation, or from expert elicitation (i.e., the process of querying, synthesizing, and encoding expert knowledge, see Garthwaite et al. 2005; Kuhnert et al. 2010; Martin et al. 2012; O'Hagan et al. 2006). For the latter, a common point of contention is whether uncertainties can or should be described by probabilities (subjective probability, see Cooke 1991) or via non‐probabilistic representations such as evidence theory, possibility theory, or interval analysis (see, e.g., Aven and Zio 2011; Baudrit et al. 2006; Helton et al. 2004) which emphasize that the uncertainty in a quantity itself may be uncertain or entirely unknown.

#### 4.2.1 Recommendation for Ecological Modellers

We recommend that uncertainty in drivers, initial conditions or model parameters should usually be quantified as probability distributions (akin to a Bayesian prior; see Section 5). Structural uncertainties, which are often represented as a list of alternative model candidates, should be weighted by probability as well. Such a fully probabilistic uncertainty quantification greatly simplifies downstream analyses such as uncertainty propagation and model inversion. The only area where non‐probabilistic uncertainties are common and, in our opinion, necessary, is when political or societal decisions are to be considered as alternative scenarios (e.g., climate change or land use scenarios).

### 4.3 Uncertainty Propagation and Projections

The aim of *uncertainty propagation*, the second step of UA, is to calculate the output uncertainty that results from the uncertainty in model components and inputs. When these model outputs describe the future, they are often referred to as predictions and in case of non‐probabilistic uncertainties (e.g., for alternative scenarios) as projections (see also Glossary).

For simple models, probabilistic uncertainty of inputs can be forwarded to outputs analytically (as, e.g., in statistical regression models). In complex models, however, uncertainty propagation must usually be done through Monte Carlo propagation or in conjunction with a sensitivity analysis. In Monte Carlo propagation, values of all uncertain factors are sampled from their uncertainty distributions and are then evaluated by simulation, resulting in a Monte Carlo ensemble of possible model outputs that can be interpreted as a probability distribution. If input uncertainties are not weighted by probabilities (this situation commonly arises when working with model ensembles, see, e.g., Dormann et al. 2018; or by alternative climate change scenarios), the output distribution is also unweighted by probability and should thus not be interpreted as a probability distribution.

#### 4.3.1 Recommendation for Ecological Modellers

When performing Monte Carlo propagation of uncertainties, different sampling schemes are available (see comparisons by Burhenne et al. 2011; Helton and Davis 2003; Renardy et al. 2021). Random sampling is easy to implement but has the problem that the tails of the distributions are rarely sampled, even though they may be crucial for tail uncertainties. Stratified sampling schemes that enforce an even sampling effort even in regions of low probability can solve this problem. A special case of stratified sampling is Latin hypercube sampling, which aims to generate more even and uncorrelated sampling distributions. Another option is quasi‐random sampling, for example, with Sobol’ sequences, which optimize for an even distribution of samples across the sampled space and thus increase efficiency. As discussed in Section 3, it is usually possible to reuse the sample generated during the uncertainty propagation for both the calculation of sensitivities and for uncertainty attribution (see next subsection). As for the sensitivity analysis, the number of samples needed to reach stable estimates of uncertainty can scale unfavourably with the number of uncertain input factors, requiring a substantial number of model evaluations to achieve stable UA estimates (see also Box S1).

### 4.4 Uncertainty Attribution

*Uncertainty attribution*, quantifying how the uncertainty in single model inputs or components contributes to the combined outcome uncertainty, is a final optional step of an UA. Uncertainty attribution is sometimes also referred to as analysis of uncertainty importance (Saltelli 2002).

Uncertainty attribution can be done by regressing the output variance against the varied input factors using any suitable statistical or machine learning method. Alternatively, as noted before, the contribution of an input or component to the outcome uncertainty is approximately proportional to its uncertainty times its sensitivity (Figure 2). Thus, uncertainty‐scaled variance‐based global SA methods can effectively be interpreted as uncertainty attribution (for an ecological example, see Huber et al. 2018). We again stress, however, that there are important conceptual differences between SA and UA, and not every global SA method is suitable for uncertainty attribution. For example, if the goal is to rank variables by their contribution to output uncertainty, it is critical to ensure that interactions are not accounted for multiple times (e.g., when summing Sobol’ total effects, where shared interactions are counted for each involved variable; Saltelli 2002).

#### 4.4.1 Recommendation for Ecological Modellers

Having performed a Monte Carlo uncertainty propagation, it is straightforward to regress the output variance against the input factors. The advantage of using a statistical model (e.g., a linear regression or ANOVA) for this purpose is the ease of interpretation, while machine learning algorithms (e.g., artificial neural networks or random forests) are better able to account for nonlinearities and interactions between input factors. A combination of methods can therefore be useful (e.g., Oberpriller et al. 2022). Alternatively, uncertainty attribution can also be done with scaled global SA methods (e.g., Huber et al. 2018).

## 5 Model Inversion: Inverse Uncertainty Propagation and Calibration

Sensitivity and uncertainty analysis are often classified as examples of *forward modelling* or the *forward problem*. Here, ‘forward’ refers to calculating the consequences of model components (structure, parameters) and inputs (drivers, initial conditions) on model outputs. The *inverse problem* (also known as inverse modelling or model inversion) reverses this process by searching for model structures, parameters or inputs that could have produced some observed model outputs (e.g., Tarantola 2005).

### 5.1 Goal and Scope of Model Inversion

The goal of a model inversion is to gain information about probable model inputs or components by comparing model predictions with observations. Depending on the uncertainty addressed, different names are used for this process: inverse modelling for reducing parameter uncertainty is often called *parameter estimation, calibration* or *fitting*. Reducing structural uncertainty is often called *model comparison* or *model selection*. Reducing uncertainty in states or inputs is known as *data or state assimilation*. Combinations of these variants are also possible, for example when calibrating parameters while accounting for error in model inputs (see also Bayesian melding, e.g., Hefley et al. 2013; Mason et al. 2018).

**FIGURE 5:**
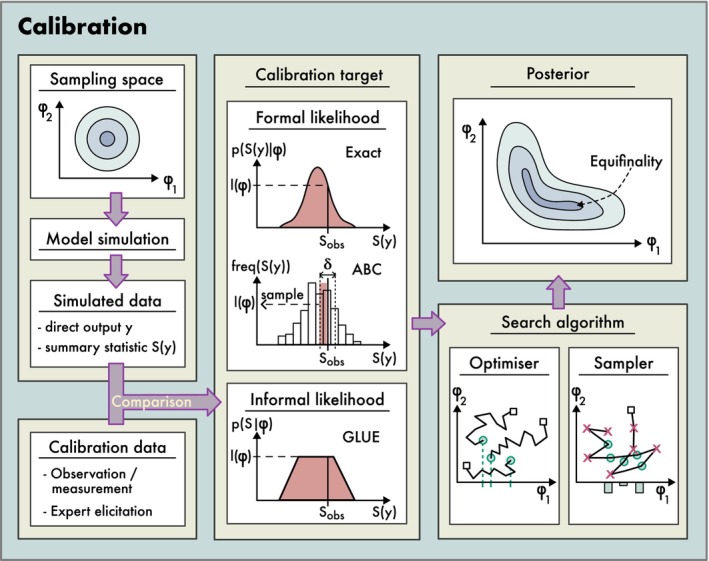
Conceptual steps in the calibration of a simulation model: The variables under calibration (parameters) φ are sampled from a defined space, for example, the prior distribution, and forwarded through the model to obtain outputs y or their summary statistics S(y). These are compared to the calibration data via a measure of fit, which is used as the calibration target. This can be a formal likelihood (exact or approximate), possibly weighted by a prior probability or an informal metric (as in GLUE or POM). Different search algorithms can then be used to scan the response surface of the calibration target. It is common that there is no unique maximum of the calibration target, meaning that the same quality of fit is reached by multiple parameterizations (equifinality).

Technically, the process of model inversion requires two key elements: (1) a calibration target consisting either of a formal statistical (probabilistic) or an informal (non‐probabilistic) metric that quantifies the fit of the model outputs to the observed data (also known as objective function, cost function, or discrepancy measure); and (2) a search algorithm that explores the response surface of this calibration target within the space defined by model uncertainty (Figure 5; see also Hartig et al. 2012). Depending on the calibration goal and philosophy, the search algorithm may either approximate the entire response surface as a function of the model components or inputs, or it identifies just the single option that produces the best fit.

**FIGURE 6:**
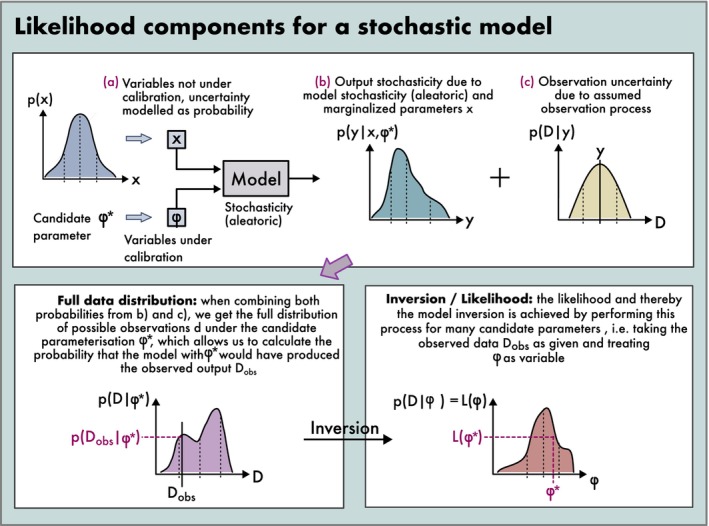
Construction of a formal likelihood function for a stochastic simulation model. The likelihood L(φ*) of a parameterization φ* is the probability of obtaining the observed data D_obs_ under this parameterization. This probability may arise from various sources of uncertainty: (a) uncertain inputs that are not included in the calibration, (b) model stochasticity, (c) data uncertainty (observation error). Together, these contributions make up the full data distribution under φ*, from which L(φ*) can be read off.

### 5.2 The Calibration Target: How to Measure Fit to the Data?

When deciding on a metric to quantify model fit, a key distinction is between probabilistic (statistical) and non‐probabilistic (informal) approaches. This choice is of fundamental importance because once a non‐probabilistic measure of fit is used, the results of the inversion will also be non‐probabilistic, rendering much of the previous machinery for uncertainty propagation and attribution inapplicable.

Probabilistic calibration methods (Bayesian or frequentist) define ‘fit’ mainly by the probability *p*(*D*|M(φ, …)) of observing the data *D* under the given model M with parameterization φ and, optionally, other uncertain model components or inputs (here denoted by ‘…’). This metric is known as the *likelihood*. It can be used, for example, to quantify how much more probable it is to obtain the observed data under parameter φ^1^ compared to parameter φ^2^. The likelihood can either be used directly as a calibration target (known as *maximum likelihood estimation*) or as a factor in *Bayes' formula*, which calculates the posterior probability of alternative model parameterizations:

$$p\left(M\left(\varphi ,\ldots \right)|D\right)=\frac{\;p\left(D|M\left(\varphi ,\ldots \right)\right)\cdot p\left(M\left(\varphi ,\ldots \right)\right)}{p\left(D\right)}$$

Bayes' formula states that the posterior uncertainty for any combination of model components and inputs under consideration is proportional to the likelihood *p*(*D*|M(φ, …)) and the prior probability $p\left(M\left(\varphi ,\ldots \right)\right)$. The prior summarizes the uncertainty about the calibration target before considering the data D. The quantity p(D) is a constant that normalizes the posterior integral to one. Intuitively, Bayes' formula updates the prior uncertainty by propagating the information in the data backward through the assumed data‐generating process (encoded in the likelihood) to the model's inputs and components (e.g., van Oijen et al. 2005). Because uncertainty is propagated backwards, this approach is also referred to as the method of ‘inverse probability’.

A limitation of Bayes' formula is that it relies on sensible definitions of the likelihood and the prior, which is not always straightforward in complex models. Because of the strong nonlinearity and high interconnectedness in complex ecological models, structural errors in the likelihood can be even more consequential than for statistical models (Oberpriller et al. 2021; Pernot and Cailliez 2017). This is particularly the case when likelihoods are based on multiple unbalanced data streams (Cameron et al. 2022).

When a model produces deterministic or quasi‐deterministic outputs, likelihoods can be constructed and checked according to the same principles used in statistical regression models. For example, normal or Poisson distributed errors around the simulated output may be assumed (e.g., van Oijen et al. 2005; Hartig et al. 2012), and more complex error structures such as correlated, heteroscedastic or skewed error distributions can be added when needed (e.g., Schoups and Vrugt 2010). If multiple data streams (multiple constraints) are available, those can be combined into a joint likelihood (Hartig et al. 2012).

When model outputs are stochastic, more advanced methods may be required. For small levels of stochasticity, outputs can be approximated as quasi‐deterministic and the former approach can be maintained. Also, output stochasticity can be reduced by averaging over several model runs (e.g., Malchow et al. 2024). With high levels of stochasticity, however, it is more appropriate to model the likelihood generated by the stochastic process itself (see Figure 6 and Box 2, ‘Likelihood‐free inference’).

BOX 2 **Likelihood‐Free Inference.**

If model outputs are highly stochastic, it is reasonable to assume that discrepancies between observations and model outputs are largely explained by this internal stochasticity rather than an external observation error. In other words, due to the stochasticity, the process‐based model itself is already a likelihood p(D|M) which can explain discrepancies between model and data and can be used as a calibration target (Hartig et al. 2011).

The problem in such a setting is that this “internal” likelihood p(D|M) of a stochastic model can typically neither be calculated analytically nor directly approximated numerically with reasonable computational effort. Such likelihoods are called *intractable*.

In such cases, approximate likelihoods or posteriors can be generated from simulations of the stochastic model using methods such as Approximate Bayesian Computation (ABC, see Beaumont 2010; Csilléry et al. 2010; Hauenstein et al. 2019; McKinley et al. 2018; Stumpf 2014) or synthetic likelihood (e.g., Hartig et al. 2014; Price et al. 2018; Wood 2010). These methods are collectively referred to as likelihood‐free inference or simulation‐based inference. In line with Cranmer et al. (2020), we prefer the latter, as the term likelihood‐free may mislead the reader into thinking that no likelihood exists, when in fact it is only intractable. For in‐depth reviews of simulation‐based inference, see Cranmer et al. (2020) and Hartig et al. (2011).

Arguably the most popular method of simulation‐based inference is ABC. It requires defining a distance δ(*D*_sim_, *D*_obs_) that measures the discrepancy between the simulated data *D*_sim_ and the calibration data *D*_obs_. Then, an approximation of the posterior *p*_ε_(φ|*D*_obs_) is obtained (e.g., using MCMC) by computing the distance δ(*D*_sim_, *D*_obs_) instead of the likelihood for each candidate parameter set φ. A proposal φ is accepted as a sample from *p*_ε_(φ|*D*_obs_) if δ(*D*_sim_, *D*_obs_) is smaller than a specified acceptance level ε. It can be shown that this approximation converges to the true posterior as the value of ε tends to zero.

It is critical to choose the acceptance level ε appropriately. A smaller ε leads to a better approximation, but also to a lower acceptance probability, which means that more simulations must be performed. Another important factor is the dimensionality of the summary statistic. The more dimensions it has, the more noise accumulates in the distance metric δ, reducing the acceptance probability. To counteract this, most ABC procedures use dimension reduction techniques on the data (Blum et al. 2013).

A new approach in the field of simulation‐based inference is the use of machine learning or deep learning models to predict parameters based on the data (e.g., Cranmer et al. 2020). These models are expected to handle high‐dimensional, complex observations more effectively, potentially eliminating the need for dimension reduction in traditional ABC techniques.

A common point of contention regarding the use of Bayes' formula is the specification of the prior, which has been criticized as being subjective (for details on this debate, see Ellison (2004) and van Zyl (2018)). So‐called informative priors can be obtained from measurements or existing literature (Kass and Wasserman 1996; Lemoine 2019) as well as expert knowledge (prior elicitation; Falconer et al. 2022; Kuhnert et al. 2010), which is essentially equivalent to the uncertainty quantification discussed in the previous section. More challenging is the definition of uninformative priors that express no prior knowledge (e.g., van Dongen 2006; Banner et al. 2020). We nevertheless view Bayes' formula as the most natural way to approach the problem of model inversion for complex ecological models because it formalizes and unifies both the a priori quantification of uncertainties and their reduction through observation of model outputs. Note that Bayes' formula can be applied even if no observations are available, only expert knowledge about model outputs (e.g., Hartmann et al. 2020).

The main alternative to likelihood‐based statistical calibration targets are informal metrics of model fit that compare the simulated model output to the calibration data without necessarily representing a probability distribution. Informal metrics are usually chosen ad hoc, although some guidelines exist that advise on how to choose appropriate informal calibration metrics. Two well‐known examples of such guidelines are generalized likelihood uncertainty estimation (GLUE; Beven and Binley 1992; Beven and Binley 2014; Mirzaei et al. 2015) in the field of hydrology and pattern‐oriented modelling (POM; Grimm et al. 2005) in ecology. In essence, GLUE promotes the use of informal fit metrics as a replacement of the likelihood in a Bayesian framework (see Smith et al. 2008; see Box 3 on the debate of whether such informal likelihoods are useful). POM proposes the use of summary statistics, ideally at different scales and organizational levels, as a set of dichotomous tests that the model needs to pass to be accepted (Grimm and Railsback 2012; for an example see Fordham et al. 2022). Both frameworks allow considerable flexibility in the design of the informal likelihood, that is, how fit is measured and how summary statistics are weighted (K. Beven 2006; Gallagher et al. 2021).

BOX 3 **The Formal vs. Informal Likelihood Debate.**

The virtues and limitations of formal and informal objective functions have sparked many debates, as exemplified by a prominent exchange of papers (K. J. Beven 2009; Vrugt et al. 2009) between Keith Beven, the originator of the informal GLUE method (K. Beven 2006) and Jasper Vrugt, who argued in favour of formal statistical calibrations.

The statistical argument (represented by Jasper Vrugt) is as follows: Assume that the model structure (including the error model represented in the likelihood) is correct, that is, that a parameterization φ* exists, that fully explains the calibration data up to the measurement error and model stochasticity. Then, it can be shown that statistical inversion procedures such as the MLE or the Bayesian posterior deliver the best possible reduction of uncertainty, given the data. No such guarantee exists for informal calibrations, and thus informal objectives likely produce sub‐optimal estimates of model parameters and their uncertainties. The reason for this is that guessing an optimal goodness‐of‐fit measure is inherently difficult without having a formal system that guides this decision.

Proponents of informal objectives (represented by Keith Beven) argue that the assumption encoded in the likelihoods rarely hold for complex models in practical situations. Yet, if there is structural error, none of the above guarantees are valid and inferences can be seriously wrong (e.g., Oberpriller et al. 2021). In such cases, informal objectives (Smith et al. 2008), guided by the experience of the modeller, the specific objectives of the modelling project, and the guidelines from frameworks such as generalized likelihood uncertainty estimation (GLUE) or Pattern‐oriented modelling (POM) may work better (Beven and Smith 2015).

To summarize the debate: Statistical approaches assume that data variation arises only from stochasticity (aleatoric error), while informal approaches such as GLUE assume a mostly epistemic error. If the error is mainly stochastic, statistical approaches likely provide better inferences because they generate probabilistic uncertainties. Informal approaches may be more robust in the face of structural error (Nourali et al. 2016); however, their main limitation is that they generate possibilistic (rather than probabilistic) uncertainties.

Our own position is that informal approaches can be considered when structural model error is thought to be dominant. This may lead to better inference and makes it explicit that the resulting uncertainties are non‐probabilistic. Nevertheless, we believe that the aspiration of modellers should be to reduce structural error and, wherever possible, apply formal statistical approaches for stronger inference.

### 5.3 The Search Algorithm

Once the calibration target is selected, its response surface must be explored over the space of uncertain model components and/or model inputs. Algorithms for model inversion can be distinguished by their objective: optimization algorithms seek the global optimum (minimum or maximum), while Markov Chain Monte Carlo and similar algorithms seek to approximate the shape of the response surface. For either of these algorithms, runtime and scalability to high‐dimensional search spaces (see Glossary: ‘curse of dimensionality’ and Box S1) is a key design criterion.

For optimization, many strategies exist, including gradient‐based (local) algorithms and evolutionary (global) algorithms (e.g., Mullen et al. 2011). When an optimum is found, the shape of the response surface around this optimum can be approximated, thus constructing a confidence interval or similar measures of uncertainty about the true optimum. A disadvantage of optimization approaches is that they can easily get stuck in local optima, and that, due to limited data and high model complexity, there may be multiple optima or ridges (trade‐offs) in the response surface, leading to parameter non‐identifiability, also known as equifinality (Glossary).

Algorithms that estimate the full response surface, rather than searching for an optimum, are therefore often preferable for complex ecological models. The most common algorithms of this sort either use Monte Carlo methods that concentrate sampling effort on areas of high target density, or semi‐analytical approaches such as variational inference (Blei et al. 2017). Monte Carlo methods include Markov Chain Monte Carlo (MCMC) and Sequential Monte Carlo (SMC), which include particle filters (Doucet and Johansen 2011; Speich et al. 2021). While MCMC methods evaluate an acceptance step for each proposal, SMC iteratively weights and resamples a set of proposals (for a methodological overview and comparison, see Luengo et al. (2020) and Speich et al. (2021)).

### 5.4 Recommendations for Ecological Modellers

As noted above, we generally recommend using probabilistic representations of uncertainty where possible, and we consider a full Bayesian calibration, based on a probabilistic quantification of prior uncertainties and model fit (via the likelihood) to be the gold standard for model inversion (for a classic ecological example, see e.g., the Bayesian calibration of a forest model by van Oijen et al. 2005).

In such a setting, prior distributions should be chosen based on a systematic assessment of model uncertainties (see Section 4). If parameters have a direct ecological meaning, priors can and should consider domain knowledge and direct measurements (Hartig et al. 2012; Banner et al. 2020). Otherwise, uninformative priors must be chosen, keeping in mind that these are not necessarily uniform (flat) and must be selected with care (e.g., van Dongen 2006). In any case, the implications of the prior assumptions should be checked using prior sensitivity analyses and prior predictive checks (e.g., Wesner and Pomeranz 2021).

An important concern in model inversion is the bias and variance trade‐off. It states that, when high model flexibility is met with little data, calibration may lead to overfitted models that generalize poorly to new data. To detect this problem, it is useful to test the model's predictions on independent data that was not used in the calibration process (e.g., Cailleret et al. 2020 for an ecological example). If overfitting is detected, generalization can often be improved by using so‐called regularizing priors (Lemoine 2019). These priors intentionally trade off a reduction in the complexity of the inversion problem against a small bias in the parameter estimates. Regularizing priors are not designed to fully reflect all uncertainties in model components or inputs. However, with limited data, they can still result in better predictions and should thus be considered when data is scarce.

Although optimization algorithms often require fewer model evaluations, we believe that algorithms that explore the entire response surface (e.g., MCMCs) are preferable for complex inversion problems for the reasons explained above. Population MCMCs (such as the DEzs algorithm (ter Braak and Vrugt 2008)) are particularly suitable for complex models because they can be partially parallelized and do not require the ability to compute model derivatives. The choice of algorithm also depends on the objective function (i.e., the measure of fit) and the calibration target. Binary objectives, which are often used in POM, are readily sampled within a Latin hypercube scheme (Helton and Davis 2003; as an example, see Fordham et al. 2022), whereas data assimilation is usually addressed with SMC (cf. Speich et al. 2021).

Identifiability analysis (IA) can often be useful in complementing the calibration process. IA assesses how well model parameters can be estimated from the available set of inputs and outputs (e.g., Guillaume et al. 2019). A simple approach to IA is to perform a SA on the inversion target: Parameters that influence the target more strongly also tend to be more identifiable from the information in the calibration data. The concept of model sloppiness (e.g., Monsalve‐Bravo et al. 2022) formally extends this idea. Another important check is calibrating the model to synthetic data that was generated by the same model to see if the parameter values are recovered correctly (for an ecological example, see Hartig et al. 2014).

Our final comment concerns the computational cost of inversion methods, which can be substantial for complex models, especially when estimating the full shape of the calibration target (cf. Box S1). To mitigate this problem, one option is to find a more efficient search algorithm, which is often possible. A second option is to parallelize the model or the calibration algorithms and leverage modern parallel computing environments. A third option is to speed up model execution, either by optimizing the model code or by using surrogate models (emulators) to approximate the model output or the calibration target (e.g., Craig et al. 2001; Dietzel and Reichert 2014). Emulation via machine learning and deep learning approaches can also be considered (Lamperti et al. 2018).

## 6 Evaluating and Communicating Prediction and Decision Uncertainty

In this final section, we consider the practical implications of the methods discussed, both regarding their relevance for downstream analyses and predictions in ecology, and regarding their value in providing relevant information to the scientific community and society.

**FIGURE 7:**
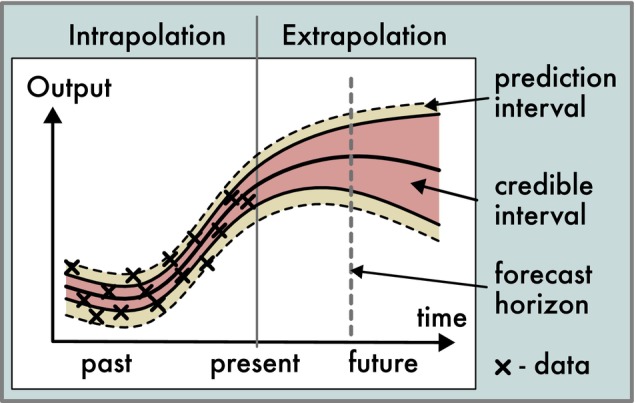
Example of an uncertainty visualization for the temporal predictions of a dynamic model that was calibrated to observed reference data, marked by crosses (interpolation region). The central solid line shows the median prediction; the red area shows the credible interval (i.e., the part of the prediction uncertainty that can be attributed to uncertain model components), and the yellow plus red area depicts the prediction interval (i.e., combined uncertainty of the observation process that is encoded in the likelihood and the parameter uncertainty represented by the credible interval). The visualization also highlights that predictions are extended into an extrapolation region where no data is available, potentially introducing additional deep extrapolation uncertainty that is difficult to quantify.

First, as noted in many other reviews, the treatment of uncertainties should encompass all relevant uncertainties in a consistent way (Bodner et al. 2020; Zylstra and Zipkin 2021). While this may sound obvious, many practical studies still consider only one type of uncertainty (e.g., parameter uncertainty). Once uncertainties are fully described, the domain for which the model can make sufficiently precise predictions should be explored. For example, the forecast horizon describes the time at which a given maximum of accepted prediction uncertainty is exceeded (Figure 7; Petchey et al. 2015). A forecast horizon can also be determined for extrapolations in other dimensions, such as space or environmental conditions, to establish the domain of applicability of a forecast. It should be noted, however, that extrapolating in space and time introduces additional deep uncertainty that is often difficult to quantify (Spiegelhalter and Riesch 2011).

In many cases, model predictions will be integrated into a larger decision‐making process, which could be based on cost–benefit analysis (Mishan and Quah 2020), adaptive management (Westgate et al. 2013) or similar frameworks. All techniques discussed in this article provide crucial inputs for these methods. For example, a probabilistic model calibration may reduce uncertainty on the economic consequences of certain management decisions, which can then aid decision making (for an example see Augustynczik et al. 2017). In this setting, discrepancies may arise between the data that is most helpful to reduce model uncertainties vs. the data that best reduces decision uncertainties. Frameworks such as the Value of Information (VOI; e.g., Bolam et al. 2019) can guide observation and calibration efforts for reducing decision‐making uncertainty.

A final step in forecasting and prediction‐making is the effective communication of the results. Many guidelines exist for both the verbal expression (e.g., Bodner et al. 2020; Spiegelhalter 2017) and visualization (e.g., Franconeri et al. 2021) of uncertain results. We recommend that visualizations of model predictions should include credible intervals, prediction intervals, and an indication of the regions of interpolation and extrapolation (e.g., by displaying the region for which data is available, see Figure 7). If it was not possible to cover all major sources of uncertainty in the analysis, this should be clearly communicated. Moreover, a discussion of the level and location of uncertainties (see Section 2.1) is advisable, illustrating what information was used to determine the chosen representation of uncertainty and the modeller's confidence in it (Kandlikar et al. 2005). Overconfident uncertainty quantifications can lead to misleading results or potentially be misused to influence opinions and decisions.

## 7 Discussion and Conclusion

Ecological models are often used to inform critical management decisions for complex systems with limited data. A consistent treatment of uncertainty is central to their credibility. In this review, we placed sensitivity analysis, uncertainty analysis and inverse modelling into a common framework to explore, understand, and propagate uncertainties in ecological models and to assess their implications for predictions. While a complete uncertainty assessment ideally incorporates all these methods, applying even one of them can already generate valuable insights about the ‘uncertainty landscape’ of a modelling project.

One insight from this review is that despite the long history of the field, there is still considerable controversy about best practices. This is largely due to the high diversity of methods, approaches and concepts scattered across different disciplines. To help the reader navigate this diversity, we have provided recommendations for each methodological approach, while acknowledging that many of these recommendations are based on our practical experience rather than comprehensive methodological comparisons.

Many open questions remain. For instance, a current topic in statistics is the integration of large heterogeneous datasets. This typically requires multiple observation models (so‐called integrated statistical models) to combine the different data streams for model inversion. For process‐based models, how to integrate multiple data streams while at the same time dealing with structural uncertainty remains a challenge (e.g., Oberpriller et al. 2021). Another current trend is the combination of process‐based models with AI and ML methods for uncertainty propagation, simulation‐based inference, parameterization, physics‐informed ML and emulators.

Notwithstanding these future developments, uncertainty quantification and reduction will remain essential for the application of complex models to questions of societal importance. We hope that this review will provide guidance for practical applications and inspire new research in this exciting and important area of computational research.

## Author Contributions

Anne‐Kathleen Malchow and Florian Hartig jointly conceived and designed the study. They contributed equally to the writing and preparation of the manuscript. Both authors revised and approved the final version of the manuscript. The authors declare no conflicts of interest.

## Funding

This work was supported by the Bavarian State Ministry for Science and the Arts, bayklif.

## Supporting information


**Table S1:** Overview of selected methods for sensitivity analysis. Methods are grouped into local, semi‐local (or one‐at‐a time; OAT) and global methods (row colours) according to the part of the input factor space that is sampled (first column). For each method, the sensitivity measure, a short description, central properties and a relevant reference are given (columns 2–5).

## Data Availability

No data were generated or analyzed for this review article.
