# Supplementary material for: Calibration, Sensitivity and Uncertainty Analysis of Complex Ecological Models—A Review
Source: Ecol Lett. 2026 Apr 26;29(4):e70375. doi: 10.1111/ele.70375 (PMC13110421; doi:10.1111/ele.70375)
Supplement: Supplementary file 1 — Table S1: Overview of selected methods for sensitivity analysis. Methods are grouped into local, semi‐local (or one‐at‐a time; OAT) and global methods (row colours) according to the part of the input factor space that is sampled (first column). For each method, the sensitivity measure, a short description, central properties and a relevant reference are given (columns 2–5). [file ELE-29-0-s001.pdf]

Supporting information for

Anne-Kathleen Malchow & Florian Hartig: Calibration, sensitivity and uncertainty analysis of complex ecological models – a review

TABLE S1 Overview of selected methods for sensitivity analysis. Methods are grouped into local, semi-local (or one-at-a time; OAT), and global methods (row colors) according to the part of the input factor space that is sampled (first column). For each method, the sensitivity measure, a short description, central properties, and a relevant reference are given (columns 2-5).

	Method	Sensitivity measure	Description	Properties	Reference
local	Derivatives	Local slope	Calculates the change in output resulting from an infinitesimal deviation of the inputs from a reference value x^*; equals the gradient of the response surface at x^*.	Usually only the first derivative is used (linear slope), but higher orders and interactions are possible.	Reviewed in (Borgonovo and Plischke, 2016)
local	Finite differences	Non-local slope	Calculates the absolute or relative change in model output in response to a per-input deviation from x^* by a given finite amount (e.g. ±10%); corresponds to the slope between the two points.	Includes non-linear effects and is more robust than the local derivative.	Reviewed in (Borgonovo and Plischke, 2016)
semi-local (OAT)	Response curve (sometimes called spider plot)	Response over input range	Shows the absolute or relative change in model output in response to different amounts of deviation from a single input from x^*.	Provides a visual assessment of sensitivity over the range of input values.	(Eschenbach and McKeague, 1989; Saraiva et al., 2017)
semi-local (OAT)	Accumulated local effects (ALE) plot	Locally adjusted sensitivity slopes	Divides the input range into intervals and computes the average sensitivity using only data points falling within that interval. Then accumulates these local effects across the entire input range.	Provides a visual assessment of direct effect sensitivities. Does not require inputs to be uncorrelated.	(Apley and Zhu, 2020)
	Morris screening	Summary statistics of repeated finite differences across the input space	Finite differences are calculated along multiple trajectories by sequentially varying each input; resulting values are summarized by mean (quantifies average effects) and SD (quantifies	Computationally efficient by requiring fewer samples than other global methods, however its stochastic algorithm may cause convergence issues; gives qualitative	(Morris, 1991; Paleari et al., 2021)

global			interactions and nonlinearities).	rather than quantitative results (screening)	
	Derivative-based global sensitivity measures (DGSM)	Summary of local slopes	Partial derivatives are calculated over the full parameter space and summarized by mean, SD, and quantities derived from them.	More accurate than Morris, especially for non-monotonic targets. Gives upper bound on the total-order Sobol' index S_T of each input while requiring comparatively few samples.	(Kucherenko et al., 2009)
	Fourier amplitude sensitivity test (FAST)	Variance	Full decomposition of output variance based on the coefficients of the Fourier series of the output function; in theory all partial contributions can be computed, in practice usually only direct effects S_1.	Efficient method for first-order variance-based sensitivity indices; requires continuous output function; assumes uncorrelated predictors	(Saltelli and Bolado, 1998)
	Sobol' method and ANOVA	Variance	Full decomposition of output variance into all orders of input variances; in theory all partial contributions can be computed, in practice usually only direct and total effects, S_1 and S_T.	Provides first-order and total-order variance-based sensitivity indices; assumes uncorrelated predictors; allows discontinuous output; requires comparatively many samples	Sobol': (Sobol', 1990; Sobol', 2001), application of ANOVA: (Ginot et al., 2006)
	Distributed evaluation of local sensitivity analysis (DELSA)	Local variance	Locally approximates first-order Sobol' indices using a decomposition of the so-called prediction variance which is based on partial derivatives.	Hybrid local-global method designed to efficiently reveal how sensitivity varies across the parameter space	(Rakovec et al., 2014)
	Regression model (Surrogate model)	Correlation coefficients	The response surface is approximated with a linear regression model and its (standardized) regression coefficients of different orders are interpreted as sensitivities.	The form of the regression model determines which sensitivities are calculated. In practice, first and second order effects are typically used	(Kleijnen, 1992)
	Machine learning model, e.g. random forest (RF) or artificial neural network (ANN) (Surrogate model)	Variable importance	The response surface is approximated with a machine learning model and its variable importances are interpreted as sensitivities, e.g. random forest importance or SHAP for ANNs.	Model-free (captures nonlinearities and interactions). Variable importances effectively measure total effects, but specialized methods exist for higher-order effects	RF: Harper et al. (2011); ANN: Schröder et al. (2020)

REFERENCES Table S1

Apley, D.W., Zhu, J., 2020. Visualizing the Effects of Predictor Variables in Black Box Supervised Learning Models. J. R. Stat. Soc. Ser. B Stat. Methodol. 82, 1059–1086. https://doi.org/10.1111/rssb.12377

Borgonovo, E., Plischke, E., 2016. Sensitivity analysis: A review of recent advances. Eur. J. Oper. Res. 248, 869–887. https://doi.org/10.1016/j.ejor.2015.06.032

Eschenbach, T.G., McKeague, L.S., 1989. Exposition on Using Graphs for Sensitivity Analysis. Eng. Econ. 34, 315–333. https://doi.org/10.1080/00137918908902996

Ginot, V., Gaba, S., Beaudouin, R., Aries, F., Monod, H., 2006. Combined use of local and ANOVA-based global sensitivity analyses for the investigation of a stochastic dynamic model: Application to the case study of an individual-based model of a fish population. Ecol. Model. 193, 479–491. https://doi.org/10.1016/j.ecolmodel.2005.08.025

Harper, E.B., Stella, J.C., Fremier, A.K., 2011. Global sensitivity analysis for complex ecological models: a case study of riparian cottonwood population dynamics. Ecol. Appl. 21, 1225–1240. https://doi.org/10.1890/10-0506.1

Kleijnen, J.P.C., 1992. Sensitivity analysis of simulation experiments: regression analysis and statistical design. Math. Comput. Simul. 34, 297–315. https://doi.org/10.1016/0378-4754(92)90007-4

Kucherenko, S., Rodriguez-Fernandez, M., Pantelides, C., Shah, N., 2009. Monte Carlo evaluation of derivative-based global sensitivity measures. Reliab. Eng. Syst. Saf., Special Issue on Sensitivity Analysis 94, 1135–1148. https://doi.org/10.1016/j.ress.2008.05.006

Morris, M.D., 1991. Factorial Sampling Plans for Preliminary Computational Experiments. Technometrics 33, 161–174. https://doi.org/10.1080/00401706.1991.10484804

Paleari, L., Movedi, E., Zoli, M., Burato, A., Cecconi, I., Errahouly, J., Pecollo, E., Sorvillo, C., Confalonieri, R., 2021. Sensitivity analysis using Morris: Just screening or an effective ranking method? Ecol. Model. 455, 109648. https://doi.org/10.1016/j.ecolmodel.2021.109648

Rakovec, O., Hill, M.C., Clark, M.P., Weerts, A.H., Teuling, A.J., Uijlenhoet, R., 2014. Distributed Evaluation of Local Sensitivity Analysis (DELSA), with application to hydrologic models. Water Resour. Res. 50, 409–426. https://doi.org/10.1002/2013WR014063

Saltelli, A., Bolado, R., 1998. An alternative way to compute Fourier amplitude sensitivity test (FAST). Comput. Stat. Data Anal. 26, 445–460. https://doi.org/10.1016/S0167-9473(97)00043-1

Saraiva, J.P., Lima, B.S., Gomes, V.M., Flores, P.H.R., Gomes, F.A., Assis, A.O., Reis, M.R.C., Araújo, W.R.H., Abrenhosa, C., Calixto, W.P., 2017. Calculation of sensitivity index using one-at-a-time measures based on graphical analysis. 2017 18th Int. Sci. Conf. Electr. Power Eng. EPE 1–6. https://doi.org/10.1109/EPE.2017.7967329

Schröder, L., Dimitrov, N.K., Sørensen, J.A., 2020. Uncertainty propagation and sensitivity analysis of an artificial neural network used as wind turbine load surrogate model. J. Phys. Conf. Ser. 1618, 042040. https://doi.org/10.1088/1742-6596/1618/4/042040

Sobol′, I.M., 2001. Global sensitivity indices for nonlinear mathematical models and their Monte Carlo estimates. Math. Comput. Simul. 55, 271–280. https://doi.org/10.1016/S0378-4754(00)00270-6

Sobol', I.M., 1990. On sensitivity estimation for nonlinear mathematical models. Mat. Model. 2, 112–118.

Box S1: On the curse of dimensionality and the scaling of SA, UA and inversion algorithms with the number of model parameters

The curse of dimensionality (Bellman, 1957, see also glossary) refers to the fact that the points needed to cover a *d*-dimensional Euclidean space grow exponentially with *d,* while the relative volume of any subset of this space goes to zero. Despite being a trivial consequence of the geometry of Euclidean space, this behavior can have unintuitive and often problematic consequences for applications in statistics and machine learning (see Aggarwal et al., 2001; Giraud, 2021).

To understand why these issues arise, assume, for example, that we want to conduct an SA, UA or a calibration for a model with 100 parameters. If we tried to cover the model's parameter space with a regular n-dimensional grid with 10 values per dimension, we would require 10^100 model evaluations. This is far larger than the number of model evaluations that can be done in practice, which will be in the order of 10^6-10^9.

Even more disconcerting, however, is that if the area of "relevant" parameter values for a given calculation is concentrated along 1/3 of the parameter space in each dimension (e.g. because the model is only sensitive for these selected parameters), the associated "relevant" parameter volume becomes miniscule with 1/3^100 ≈ 1.94 * 10^-48, with very little chance that a random parameter sample would intersect with this "interesting" space. In other words, high-dimensional spaces are huge but often mostly empty or sparse (cf. Giraud, 2021), making even simple calculations, such as a mean response, challenging.

Superficially, it seems as if this makes UA, SA, or calibrations for parameter spaces of high-dimensional models with *d*>20 or so effectively impossible. In practice, however, statisticians regularly calibrate models with >1000 parameters using optimization and MCMC algorithms. The first reason for this is that we are usually not interested in the full *d*-dimensional model response surface, but rather in low-dimensional statistics that are calculated across this response surface. For example, when calculating the mean sensitivity across a 100-*d* parameter volume, we may already get a good approximation with 10^4-10^5 samples, provided the variance of the sensitivities across the space is not too extreme, and provided that the area of "interesting parameter space" is not too localized to a small volume (see calculation about the relative size of the "interesting" volume above).

If the volume of interesting space is too small, random sampling is inefficient, but the sparsity of the space can also be a blessing in disguise ("blessing of dimensionality", reviewed in Gorban & Tyukin, 2018) for other algorithms. As mentioned above, the number of points needed to cover a 100-d space is gigantic, but the volume of space that is "relevant" for our calculations (such as fit) can be miniscule and (via the arguments above) and may grow far slower with d. The practical problem of algorithms for exploring high-dimensional spaces is thus to find this relevant space and explore it efficiently, while neglecting the vast area of empty volume around. MCMC algorithms, for example, achieve this by randomly "walking" towards areas with higher target density while still exploring the space with kernels or algorithms that can be chosen adaptively to the current estimate of the target shape. If the response surface is sufficiently smooth and well-behaved, this can overcome or at least alleviate the curse of dimensionality (cf. Robert & Casella, 2009). Only if response surfaces are highly irregular, e.g. featuring strong non-linearities like sharp ridges

(discontinuities) and multiple optima, MCMCs may struggle to produce exact posterior shapes (Albert et al., 2025).

In practical work with process-based models, both “curse” and “blessing” of dimensionality effects occur, but not always to the same degree. As a rule of thumb, the more independently parameters act on the output under consideration, and the better the data (thus leading to localized “interesting” parameter volumes), the better will algorithms scale for models with a large number of parameters. In these cases, convergence rates of calculated indices and distributions can be nearly independent of the number of model parameters.

However, for models with strong interactions, highly nonlinear and irregular responses, and weak data constraints, it is possible that the number of calculations needed to achieve stable estimates grows exponentially with d. In such cases, the curse of dimensionality may become a problem with as little as 10 model parameters.

These insights motivate not only the development of better algorithms, but also guide the design of models: For models to work well with the methods presented here, redundant parameterizations, strong interactions, and other factors that trigger the curse of dimensionality should be avoided where possible.

References

Aggarwal, C. C., Hinneburg, A., & Keim, D. A. (2001). On the surprising behavior of distance metrics in high dimensional space. In International conference on database theory (pp. 420-434) Springer Berlin Heidelberg. https://doi.org/10.1007/3-540-44503-X_27

Bellman, R. E. (1957). Dynamic programming, Princeton University Press, Princeton, New Jersey.

Giraud, C. (2021). Introduction to high-dimensional statistics. Chapman and Hall/CRC.

Gorban, A. N., & Tyukin, I. Y. (2018). Blessing of dimensionality: mathematical foundations of the statistical physics of data. Philosophical Transactions of the Royal Society A: Mathematical, Physical and Engineering Sciences, 376(2118), 20170237. https://doi.org/10.1098/rsta.2017.0237

Albert, J., Balázs, C., Fowlie, A., Handley, W., Hunt-Smith, N., ... & White, M. (2025). A comparison of Bayesian sampling algorithms for high-dimensional particle physics and cosmology applications. Computer Physics Communications, 315, 109756. https://doi.org/10.1016/j.cpc.2025.109756

Robert, C. P., & Casella, G. (2009). Metropolis–hastings algorithms. In Introducing Monte Carlo Methods with R (pp. 167-197). New York, NY: Springer New York. https://doi.org/10.1007/978-1-4419-1576-4
